# Hazelnut Protein and Sodium Alginate Complex Coacervates: An Effective Tool for the Encapsulation of the Hydrophobic Polyphenol Quercetin

**DOI:** 10.1021/acsomega.4c04859

**Published:** 2024-08-20

**Authors:** Nabil Adrar, Fatma Duygu Ceylan, Esra Capanoglu

**Affiliations:** † Department of Food Engineering, Faculty of Chemical and Metallurgical Engineering, Istanbul Technical University, 34469 Istanbul, Turkey

## Abstract

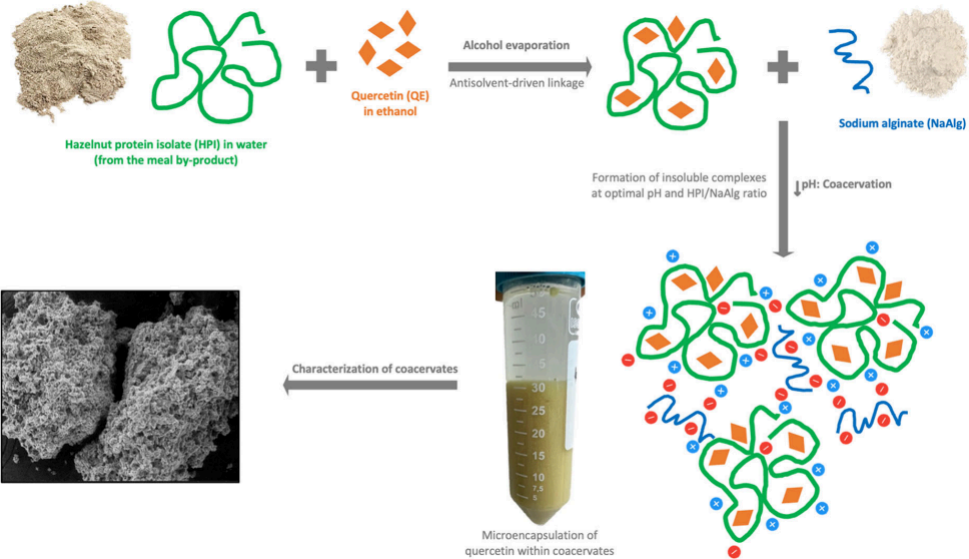

For valorization purposes of hazelnut byproducts, complex coacervation of hazelnut protein isolate (HPI) with sodium alginate (NaAlg) was investigated by turbidimetric analysis and zeta potential determination as a function of pH and protein/alginate mixing ratio. HPI-NaAlg complex coacervates were used as an encapsulating material of quercetin (QE) at different concentrations. The optimal pH and mixing ratio resulting in the highest turbidity and neutral charge were 3.5 and 6:1, respectively. The coacervation yield was 74.9% in empty capsules and up to 90.0% in the presence of QE. Under optimal conditions, HPI-NaAlg complex coacervates achieved an encapsulation efficiency higher than 99% in all coacervate/QE formulations. Fourier transform infrared spectroscopy (FTIR) results revealed the occurrence of electrostatic interactions between different functional groups within the ternary complex in addition to hydrogen and hydrophobic interactions between QE and HPI. HPI-NaAlg complex coacervates can serve as an alternative matrix for the microencapsulation of bioactive ingredients with low water solubility in food formulations, which adds an additional valorization of hazelnut byproducts.

## 1 Introduction

Microencapsulation is a process that allows the protection of bioactive compounds from environmental conditions and eventually the improvement of their bioaccessibility.^1^ Another important solution that microencapsulation offers in the food industry is taste modulation of bioactive ingredients/extracts before their incorporation in functional food formulations (e.g., bitterness and astringency masking of polyphenols), which consequently improves consumers’ acceptability for functional foods.^2^ The choice of the microencapsulation technique and wall material is crucial for effective encapsulation of ingredients, as well as to achieve the desired functionality and the stability of the capsules themselves. Factors such as the nature of the core material (e.g., molecular weight, electrical charge, solubility, melting point, volatility, sensitivity to heat and light), the intended application, and possibility of scaling up may guide the determination of which technique is the most suitable in every specific case.^3,4^

Quercetin is a flavonol, a subclass of flavonoids, found in common foods such as onions, pepper, apples, grapes, berries, tea, and nuts as well as in some medicinal plants. It is an important bioactive molecule with many reported health-promoting benefits including but not limited to antioxidant, anti-inflammatory, anticancer, antimicrobial, neuroprotective, and cardioprotective effects.^5,6^ Despite its health benefits, quercetin is known for its low bioavailability (<10%), which is due to its hydrophobic character,^7^ and its unpleasant bitter taste, which restricts its application in healthy food systems.^8^ Thus, microencapsulation may improve both bioaccessibility and organoleptic attributes of functional foods based on supplementation with bitter and hydrophobic bioactive compounds, such as quercetin.

For the specific application in the food domain, microencapsulation using the coacervation method pops up with superior advantages in comparison with other microencapsulation techniques (e.g., liposomes, spray drying, solvent evaporation, ionic gelation, interfacial polymerization, and molecular inclusion complexation) owing to its very high encapsulation efficiency (up to 99%), operating at low or ambient temperature, cost-effectiveness, and not requiring specific equipment or toxic solvents.^9,10^

Complex coacervation uses proteins as the main coating material in combination with polysaccharides for the microencapsulation of a variety of substances and molecules. This method is particularly suitable for application in the emerging functional food industry owing to the biocompatibility and low cost of the coating materials.^11^ Several protein sources were used for the formation of complex coacervation, which can be from either animal or vegetal origins. In 2020, the global production of in-shell hazelnut exceeded one million tons, from which a part is used for hazelnut oil production, leading to the generation of considerable amounts of hazelnut meal, which is still a largely underutilized industrial byproduct.^12^ It is worth mentioning that the meal is around 40% of the hazelnut kernel and is composed of 39–54% protein.^12^ Thus, hazelnut meal may emerge as a very affordable protein source for the microencapsulation of quercetin by the coacervation method.

The occurrence of complex coacervation is influenced by the charge of the two biopolymers simultaneously. The latter depends mainly on the pH and the isoelectric point of the biopolymers at other determined influencing factors, such as ionic strength, protein-to-polysaccharide ratio, and temperature. At a neutral pH, the polymers are generally co-soluble, while during acid titration, three main events are observed consecutively. The first event occurs at a pH corresponding to the first experimentally detectable increase in turbidity due to the formation of soluble complexes and is denoted as pH_C_. The second major event is marked by the formation of insoluble complexes and a large rise in turbidity (pH_φ1_). The maximum formation of complex coacervation generally occurs at a pH that is situated between the isoelectric point (pI) of the protein and the p*K*_a_ of the polysaccharide and is denoted as pH_opt_. The third event corresponds to the dissolution of the complexes at a lower pH (pH_φ2_) and results in a decrease in turbidity.^13^ Factors related to the nature of the materials, like steric interaction, hydrophobic effects, and hydrogen bonding, were also reported to influence coacervation,^14,15^ making each formulation unique and imposing an experimental determination of the optimal conditions of coacervation.

In order to valorize hazelnut oil meal byproduct, the current study explored for the first time the possibility of using hazelnut protein as a tool for the encapsulation of a hydrophobic bioactive molecule: quercetin. The study proposes optimized conditions for the formation of hazelnut meal protein–sodium alginate complex coacervation.

## 2 Materials and Methods

### 2.1 Materials and Reagents

Hazelnut (*Corylus avellana* L., Ordu, Turkey) oil meal, kindly donated by a local company, was used for the extraction of hazelnut protein. The proximate composition of the same meal was already determined in our recent study.^16^ Quercetin (≥95%) was purchased from Sigma-Aldrich (St. Louis, MO, USA), and food/pharmaceutical grade sodium alginate (KIMIKA ALGIN, standard type, grade IL-6: 40–80 mPa-s viscosity at 1% and 20 °C) was kindly provided by KIMICA Corporation (Tokyo, Japan). All other reagents were of analytical or higher grade.

### 2.2 Preparation of Hazelnut Protein Isolate

Hazelnut protein isolate (HPI) was obtained from hazelnut meal by means of alkali extraction and an isoelectric precipitation method as described in our previous study.^17^ Hazelnut meal was first subjected to further defatting and then dephenolization. Briefly, hazelnut meal was stirred in *n*-hexane overnight at a ratio of 1:4 (w/v), followed by decantation and drying in a fuming hood. Polyphenols were eliminated from the defatted meal by dissolving the latter in aqueous ethanol (70%) at a ratio of 0.15:1 (w/v) followed by successive vortexing (1 min) and sonication (15 min). After centrifugation (10 min, 4000*g*), the process of meal dephenolization was repeated twice more. The dephenolized meal was dissolved in distilled water (1:12, w/v) and then stirred for 1 h with the pH adjusted to 12 (5 M NaOH) for protein extraction. Afterward, the slurry was centrifuged (14480*g*, 10 min), the supernatant was collected, and the pellets were subjected to two further extractions. The collected supernatants were filtered (Whatman 4) to remove low-density particles. The pH was adjusted to 4.5 (5 M HCl) to allow protein precipitation. The precipitates were gathered after centrifugation (14480*g*, 5 min) and then redissolved in water with the pH readjusted to 7 before lyophilizing the proteins.

### 2.3 Preparation of HPI-NaAlg Coacervates for Quercetin Encapsulation

#### 2.3.1 Effect of Protein/Polysaccharide Ratio on the Formation of the HPI-NaAlg Complex

In order to define the optimal HPI/NaAlg ratio for the coacervation process, HPI and NaAlg solutions were mixed in eight different ratios (w/w), respectively 1:2, 1:1, 2:1, 3:1, 5:1, 8:1, 13:1, and 21:1. Sufficient HPI and NaAlg stock solutions at a concentration of 0.05% were prepared by dissolving each in distilled water. The solutions were stirred for 30 min and kept at 4 °C overnight (≈12 h) for full hydration of the biopolymers. The solutions were stirred again, and HPI/NaAlg mixtures were made in final volumes of 5 mL by mixing the appropriate volumes of HPI and NaAlg solutions. Under continuous stirring of the mixtures (300 rpm), the pH was progressively lowered to 4 units (pH/ORP meter, HI 2211, Hanna Instruments, Romania) using a HCl solution (0.05 M). Turbidity and zeta potential were the determining parameters for the ideal HPI/NaAlg ratio. This methodology was an adaptation of the literature.^18,19^ The absorbances of the mixtures and the individual biopolymers (with concentrations that correspond to each ratio) were measured at a wavelength of 600 nm using a spectrophotometer (UV-1700 PharmaSpec, Shimadzu, Japan) calibrated with distilled water to 0.000 absorbance. A Nano-ZS zetasizer (Malvern Instruments, Worcestershire, UK) was used to determine the zeta potential, with a refractive index of HPI set to 1.33 and an absorption of 0.1, at 25 °C. The ratio HPI/NaAlg (w/w) with the highest turbidity, which results in a neutral zeta potential value, and hence with maximum and stable interaction between HPI and NaAlg, was selected to study the effect of pH on the formation of the HPI-NaAlg coacervates.

#### 2.3.2 Effect of pH on the Formation of the HPI-NaAlg Complex

The effect of pH on the formation of HPI-NaAlg coacervates was studied after the selection of the optimal HPI/NaAlg ratio as 6:1. The turbidity titration method and zeta potential analysis were performed as described in the previous section (Section 2.3.1) by varying the pH from 6 to 1.5 using HCl solutions of different molarities (to minimize dilution of samples: 0.05, 0.1, 0.5, 1, and 5 M), with an interval of 0.5 units (±0.2). A NaOH solution (0.05 M) was used to limit the confidence interval to ±0.2 unit when necessary. Each pH value represented an individual sample, and the test was repeated three times.

#### 2.3.3 Quercetin Encapsulation within HPI-NaAlg Coacervates

Quercetin was chosen as a model bioactive molecule, with very limited solubility in aqueous media in an attempt to improve its bioavailability and potentially protect it from chemical degradation. HPI-NaAlg-encapsulated quercetin (HPI-NaAlg-QE) was prepared in five core:wall ratios: 0:1, 1:2, 1:4, 1:8, and 1:16. Polymer concentration was set to 1%. For each ratio, HPI (0.833 g) and NaAlg (0.167 g) were dissolved in 70 and 30 mL of distilled water. Before applying the optimized coacervation method to encapsulate quercetin, the latter was first linked to HPI using the antisolvent method as described by Xiang et al.^20^ Briefly, different amounts of quercetin (2-fold increasing from 0.0625 g to 0.5 g) were dissolved in 100 mL of ethanol each and then progressively added into HPI solutions under stirring. Ethanol was then removed by rotary evaporation. Subsequently, NaAlg solutions were added, and the mixtures were allowed to stir for 30 min. The coacervation process was triggered by adjusting the pH to 3.5 by using an HCl solution (0.1 M). Two aliquots of 75 μL were taken from each sample for zeta potential and microscope analysis. The samples were then centrifuged at 10000*g* for 30 min and 4 °C. The supernatants were kept for entrapment efficiency (EE) measurement, and the precipitate representing HPI-NaAlg-QE coacervates was freeze-dried and stored at −20 °C.

### 2.4 Characterization of HPI-NaAlg-QE Coacervates

#### 2.4.1 Zeta Potential Measurements

The first part of the collected HPI-NaAlg-QE aliquots was diluted to a 0.05% solid content with acidic distilled water (pH 3.5). The zeta potential was measured according to the same parameters described in Section 2.3.1.

#### 2.4.2 Optical Microscopy Observation

The second part of the fresh HPI-NaAlg-QE aliquots was diluted to 0.1% solid content. A drop of each sample was placed between lamina and the coverslip and observed with an upright optical microscope (Nikon Eclipse Ni-U; Nikon, Tokyo, Japan) at 40× magnification. The images of HPI-NaAlg-QE coacervates were captured by a coupled high-definition color camera (Nikon DS-Fi2) and processed by using NIS-Elements software (version 4.30, Nikon).

#### 2.4.3 Measurement of the Yields of Complex Coacervates

After the recovery of freeze-dried precipitates (Section 2.3.3), the coacervation yield (*C*_Y_) of HPI-NaAlg and HPI-NaAlg-QE coacervates was calculated according to Qiu et al.^21^ using the following equation:
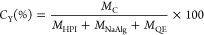
1where *M*_C_, *M*_HPI_, *M*_NaAlg_, and *M*_QE_ represent the mass (g) of the HPI-NaAlg-QE complex coacervates, HPI, NaAlg, and QE, respectively.

#### 2.4.4 Determination of Entrapment Efficiency and Loading Capacity

The entrapment efficiency and loading capacity (LC) of QE were determined by the indirect method reported by Liu et al.^22^ The concentration of free QE in the supernatants (Section 2.3.3) was measured by HPLC, and the amount of free QE in each sample was deduced from their corresponding QE concentrations and supernatant volumes. EE and LC were calculated by using the following equations:
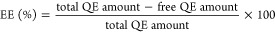
2
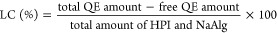
3

A Waters 2695 HPLC system equipped with a PDA (Waters 2996) detector was used to determine the QE content of HPI-NaAlg/QE coacervates. The column was a Supelcosil LC-18 (25 cm × 4.60 mm, 5 m, Sigma-Aldrich, Steinheim, Germany). Mobile A and B were Milli-Q water with 0.1% trifluoroacetic acid (TFA) and acetonitrile with 0.1% TFA, respectively. Using a linear gradient, 95% of solvent A and 5% of solvent B were used at 0 min, 65% of solvent A and 35% of solvent B were used at 45 min, and 25% of solvent A and 75% of solvent B were used at 47 min, before returning to the original conditions at 54 min. The flow rate was 1 mL/min. The detection was performed at 360 nm. UV spectra and retention times were used to identify the samples. Quantification was carried out using an external quercetin standard.^23^

#### 2.4.5 Fourier Transform Infrared Spectroscopy (FTIR) Analysis

In order to evaluate the molecular organization at the surface of the coacervates, the samples were analyzed by using an FTIR spectrometer (Bruker Tensor II) equipped with the ATR diamond module (Bruker Optics, Ettlingen, Germany). Measurements were conducted at room temperature, and each spectrum was an average of 18 scans from 4000 and 400 cm^–1^, at a resolution of 4 cm^–1^.^24^ Data were processed using Bruker Opus 7.0 FTIR and OriginPro 2022 (OriginLab, Northampton, MA, USA) software. Spectra of the coacervates were compared to those of their individual components, i.e., HPI, NaAlg, and QE.

#### 2.4.6 Differential Scanning Calorimetry Analysis

The thermal properties of the microcapsules and each individual material were examined by using differential scanning calorimetry (DSC) equipped with a cooler (Q10, TA Instruments Inc., USA). Approximately 4 mg of each dry sample was heated in tightly sealed aluminum pans from 20 to 200 °C at a heat scanning rate of 10 °C min^–1^ under a nitrogen atmosphere. An empty pan was used as a reference. Universal Analysis 2000 version 4.5A (TA Instruments Inc. USA) software was utilized for data and thermogram analysis, and the transition enthalpies (J/g) were evaluated from the area of integrated peak or dip of the plot of^25^
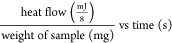
4

#### 2.4.7 Scanning Electron Microscopy (SEM) Analysis

The surface morphology of the lyophilized coacervates was examined by SEM (ThermoFisher ChemiSEM Axia). A thin layer of platinum was sputter-coated onto the samples before being photographed at a 7.5 kV acceleration voltage and a 100 μm scale with low-vacuum mode (low vacuum detector, LVD). The images were captured at a magnification rate of 1000×.

### 2.5 Statistical Analysis

Experiments were carried out in triplicate, and values were presented as means ± standard deviation (SD). Differences between means were determined by one-way analysis of variance (ANOVA) for all mean comparison except for the effect of the HPI/NaAlg ratio and pH on the optical density and zeta potential, where a two-way ANOVA test was applied. Tukey’s multiple comparison was performed as a post hoc test, and two means were considered significantly different when the *p*-value was less than the significance level of 0.05. All statistical tests were performed using GraphPad Prism 9 (San Diego, CA, USA).

## 3 Results and Discussion

### 3.1 Effect of HPI/NaAlg Ratio and pH on the Formation of Complex Coacervates

**Figure 1:**
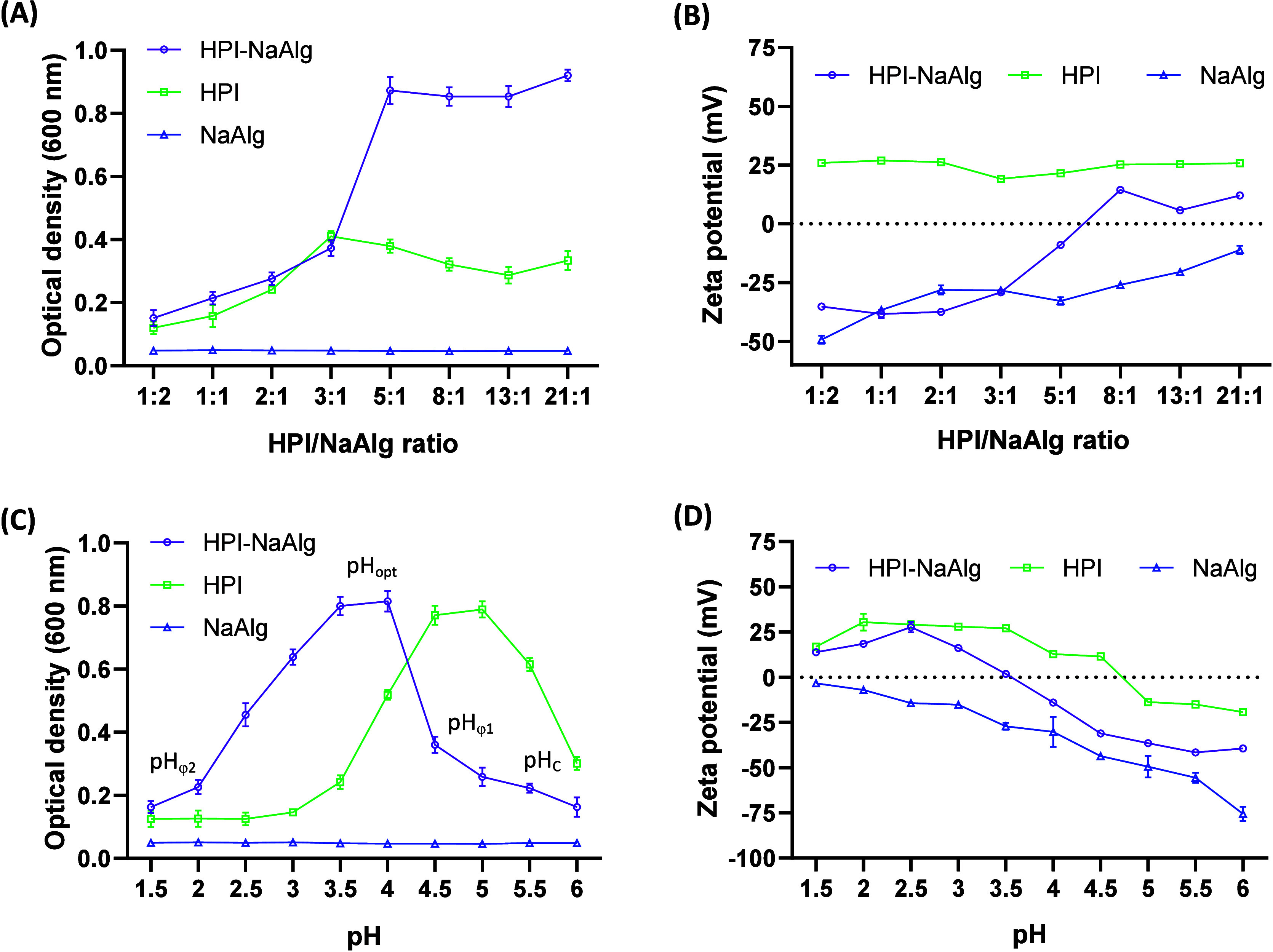
Effects of hazelnut protein isolate (HPI) to sodium alginate (NaAlg) mixing ratio and pH on the formation of HPI-NaAlg complex coacervates. A and B: impact of HPI/NaAlg ratio on, respectively, the turbidity and zeta potential at fixed pH (pH 4.0). Distilled water replaced NaAlg and HPI in the HPI and NaAlg controls, respectively. C and D: impact of pH on turbidity and zeta potential at a fixed ratio (6:1). Data represent the mean ± standard deviation (*n* = 3).

Before the optimization of pH, the optimum HPI/NaAlg ratio was determined from the turbidity and zeta potential results at a fixed pH value of 4.0. This value was chosen because the formation of protein–polysaccharide complex coacervates has been reported to typically occur between the p*K*_a_ of the polysaccharide functional groups and the isoelectric point (pI) of the protein.^19^ HPI have a pI of around 4.5,^26,27^ and the p*K*_a_ of NaAlg is in the pH range of 3.4–3.7.^28^ As illustrated in Figure 1A, the turbidity increased exponentially at the HPI/NaAlg ratio of 5:1 (optical density (OD): 0.87 ± 0.04, *p* < 0.0001) and then remained statistically unchanged until the highest ratio (*p* > 0.5). HPI controls gave positive zeta potential values at pH 4.0, which is below the pI of HPI, while NaAlg control showed negative values. HPI-NaAlg complex coacervates showed negative charges between the ratios 1:2 and 5:1, which is due to polysaccharide excess, and positive charges between the ratios 8:1 and 21:1, which is due to the increase in the protein fraction, which is charged positively at pH 4.0 (Figure 1B). The same observation was made by Qiu et al.,^21^ who obtained an increase in zeta potential values with the increase of perilla protein isolate (PPI) fraction against NaAlg at all tested pH values (pH: 2–7, PPI/NaAlg ratios: 1:1–10:1). A similar trend was obtained by Klemmer et al.^29^ for pea protein isolate and alginate polysaccharides. Interestingly, the HPI-NaAlg charge became higher than that of NaAlg only when the protein/polysaccharide ratio was higher than 3:1. This can be attributed to insufficient positively charged patches on the surface of HPI to bind all NaAlg negative functional groups, and thus one polysaccharide molecule might bind with several proteins at the same time.^30^ The charge at the surface of HPI-NaAlg stopped increasing at ratios higher than 8:1, which can indicate the saturation of NaAlg by HPI in terms of electrostatic interactions.^21^

At a pH below the pI of HPI, the balance between negatively charged carboxyl groups (−COO^–^) and positive amino groups (−NH_3_^+^) of the amino acids, which results in a neutral net charge, is altered in favor of the positive groups. Thus, a defined amount of NaAlg would restore this neutrality due to the negative carboxyl groups of NaAlg and result once again in maximum interactions between groups of the opposite charges. The HPI/NaAlg ratio with the highest turbidity that gave a neutral zeta potential was the targeted optimal ratio. While protein precipitation only requires tight bindings, which lead to their separation from water molecules, protein–polysaccharide coacervation must achieve neutrality.^31^ The curve of the zeta potential as a function of the HPI/NaAlg ratio (Figure 1B) crossed the zero line between the ratios 5:1 and 8:1. Thus, the ratio 6:1 was used to determine the optimum pH for the HPI-NaAlg complex coacervates. An optimal protein/NaAlg ratio of 6:1 was also found by Qiu et al.^21^ and Heckert Bastos et al.^19^ for perilla protein isolates and gelatin, respectively.

HPI had the highest turbidity at pH 4.5 and 5.0 (OD: 0.84 ± 0.04 and 0.89 ± 0.03) with no significant difference between these (*p* > 0.5), which corresponds to the pI of HPI (Figure 1C). Moreover, the zeta potential curve of HPI crossed the zero line within this pH range (11.80 ± 0.01 to −13.80 ± 0.26 mV) (Figure 1D). However, the highest turbidity in the HPI-NaAlg complex was found at pH 3.5 and 4 (OD: 0.80 ± 0.03 and 0.82 ± 0.03, *p* > 0.5) which is driven by the presence of anionic NaAlg. This pH range (3.5 to 4) was the optimal pH (pH_opt_) for the formation HPI-NaAlg complex coacervates, which is interpreted by the occurrence of maximum coacervation between the two biopolymers.^13^ This pH range is situated between the pI of HPI (4.5)^26,27^ and the p*K*_a_ of NaAlg (3.4–3.7).^28^ These results align with the previous report of Heckert Bastos et al.,^19^ where the formation of protein–polysaccharide complex coacervates occurred between the p*K*_a_ of NaAlg functional groups and the pI of the gelatin. The first experimentally detectable increase in turbidity occurred at pH 5.5 (pH_C_) due to the occurrence of attractive interactions between HPI and NaAlg and resulting in the first formation of soluble HPI-NaAlg complexes.^18^ There was an exponential increase in turbidity at pH 4, which was marked by the beginning of the formation of insoluble HPI-NaAlg complexes at pH_φ1_,^32^ which is pH 4.5 in the present study (Figure 1C). At low pH values (pH < 2.5), an important decrease in turbidity was observed in the HPI-NaAlg mixture; because of the low charges of NaAlg chains as well as the repulsion forces between the positively charged HPI,^33^ the HPI-NaAlg coacervates could redissolve into soluble complexes and ultimately into co-soluble and noninteracting HPI and NaAlg chains (pH_φ2_ = 2).

The zeta potential of the HPI-NaAlg complex was the closest to neutral at pH 3.5 (1.86 ± 0.34 mV) (Figure 1D). The neutralization of negative NaAlg carboxyl groups by positively charges HPI amine groups implies electrostatic binding between the two biopolymers.^32^ Moschakis and Biliaderis^34^ stated in their review that the coacervation is maximal when the complexes formed undergo charge neutralization. Another interesting observation is that at the same pH, the mean surface charges of HPI and NaAlg alone were equal to +27.10 ± 0.62 and −27.10 ± 1.91 mV, respectively, which is exactly the same value but with opposite signs. No other pH value in this study showed closer absolute values of the zeta potential between the two biopolymers. The same conclusion can be made by analyzing the results of da Silva Soares et al.^35^ in their study on ovalbumin–pectin complex coacervates. From zeta potential results, with complementary evidence from turbidity and visual microscopic aspects of the coacervates in solution as a function of pH (Supporting Information), the pH 3.5 was defined as the evident pH_opt_ for the formation of HPI-NaAlg complex coacervates in the present study. By comparing graphs A and C of Figure 1, it becomes obvious that the higher turbidity achieved by the HPI-NaAlg complex in comparison to HPI in graph A was due to the pH condition. Indeed, when each of these samples was made at its pH_opt_, there were no significant differences in the turbidity (Figure 1C).

### 3.2 Charge, Yields, and QE Encapsulation Capability of HPI-NaAlg Coacervates

HPI-NaAlg (6:1) coacervates expressed an expected neutral charge at pH 3.5 (ζ-potential = 0.27 ± 0.52) (Table 1). The presence of QE slightly increased their charge to up to 9.03 ± 0.29 mV. It was observed in our previous study that the binding to polyphenols may cause partial unfolding in hazelnut proteins.^17^ This would cause the exposure of charged groups from HPI and thus result in a slight alteration in the neutral charge. It was also reported that the binding of QE may influence the electrostatic interaction between QE-loaded protein and the polysaccharide in complex coacervates.^36^

**Table 1:** Zeta Potential, Coacervation Yields (*C*_Y_), Encapsulation Efficiency (EE), and Loading Capacity (LC) of HPI-NaAlg Coacervates^a^

Sample	Zeta Potential (mV)	C_Y_ (%)	EE (%)	LC (%)
HPI-NaAlg/QE (2:1)	9.03 ± 0.29	90.02 ± 1.26	99.94 ± 0.02	49.97 ± 0.01
HPI-NaAlg/QE (4:1)	7.24 ± 0.28	85.17 ± 0.55	99.95 ± 0.01	24.99 ± 0.00
HPI-NaAlg/QE (8:1)	5.79 ± 0.36	84.67 ± 1.80	99.94 ± 0.01	12.49 ± 0.00
HPI-NaAlg/QE (16:1)	4.47 ± 0.40	84.15 ± 1.02	99.93 ± 0.00	6.25 ± 0.00
HPI-NaAlg	0.27 ± 0.52	74.90 ± 1.33	NA	NA

a NA: not applicable. HPI: hazelnut protein isolate. NaAlg: sodium alginate. QE: quercetin.

Empty HPI-NaAlg coacervates were formed at relatively high coacervation yields (*C*_Y_) in optimal conditions (*C*_Y_ = 74.90 ± 1.33%). The presence of QE further improved the *C*_Y_ by reaching 90.0 ± 21.26% with the highest amount of QE (HPI-NaAlg/QE(2:1)) (Table 1). QE was nearly completely encapsulated regardless of the core/wall ratio used (EE around 99.94%). EE results were confirmed by the direct method after successful full release of QE in ethanol assisted by ultrasound and vigorous vortexing, which showed no significant differences (*p* > 0.05) (data not shown). The loading capacity positively and perfectly correlated with the core/wall ratio (correlation coefficient *r* = 1.00). Thus, higher QE/HPI-NaAlg ratios than 0.5 could be used, given the high EE and LC achieved in this study. According to eq 3 (Section 2.4.4), achieving a high LC means achieving a high EE with a small amount of entrapping material. It is not uncommon to achieve a high EE of hydrophobic molecules with the complex coacervation method, but the LC obtained in this study is very remarkable when compared with other studies.^22,37^ These results suggest that complex coacervates formed with hazelnut protein and alginate can be considered to be promising carriers for the encapsulation of low water-soluble bioactive compounds like quercetin to enrich beverages and a variety of food products.

### 3.3 Molecular Interactions within HPI-NaAlg-QE Coacervates

**Figure 2:**
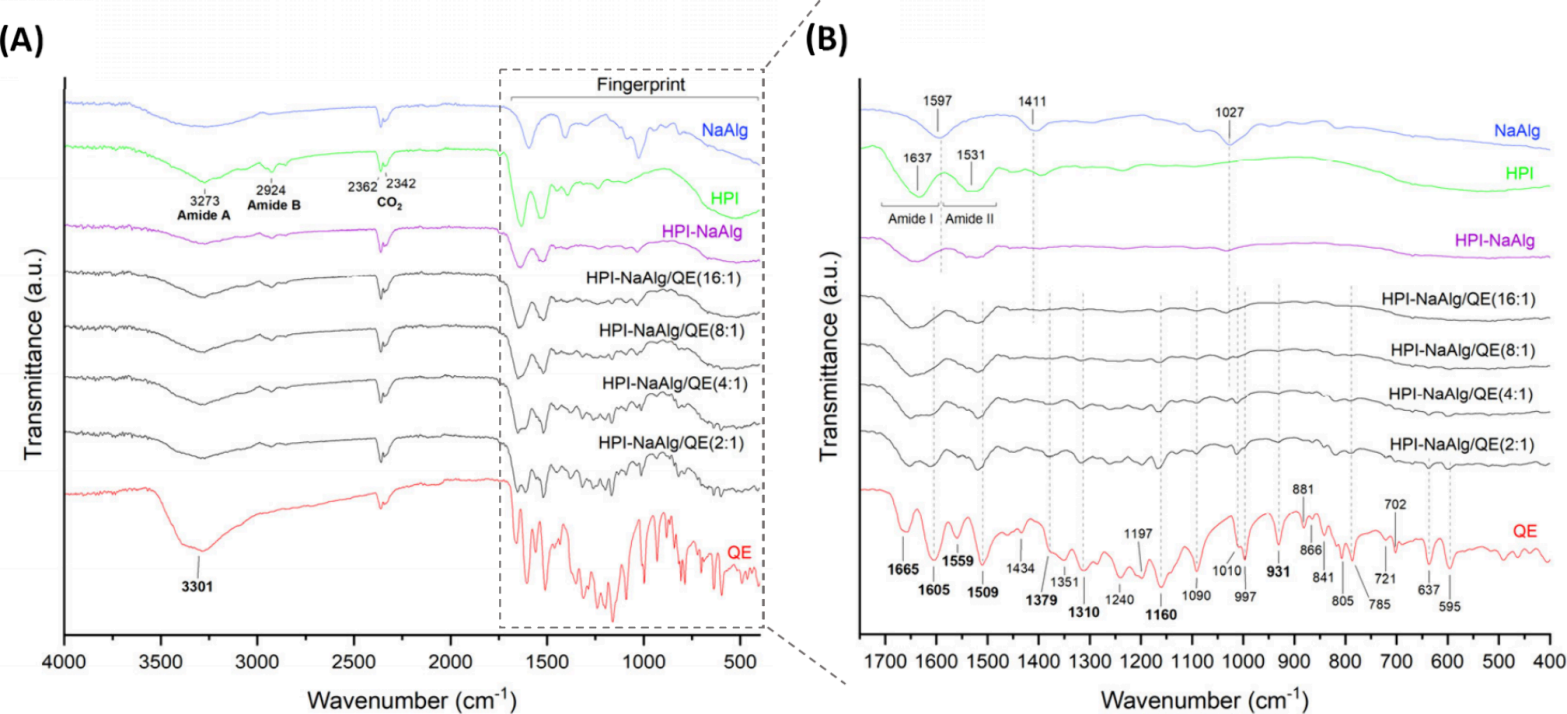
FTIR spectra of sodium alginate (NaAlg), hazelnut protein isolates (HPI), quercetin (QE), HPI-NaAlg coacervates, and HPI-NaAlg-QE microcapsules. A: Full FTIR spectra (4000–400 cm^–1^). B: Fingerprint region (1750–400 cm^–1^).

Figure 2 shows the FTIR spectra of NaAlg, HPI, HPI-NaAlg complex, and HPI-NaAlg-QE microcapsule powders analyzed in the wavelength region of 4000–400 cm^–1^. The selected wavelength region includes the N–H stretching of amide A and B bands, observed in proteins at approximately 3300 and 3100 cm^–1^, as well as the two most prominent vibrational bands of the protein backbone: amide I (1600–1690 cm^–1^) and amide II (1480–1575 cm^–1^) bands. The amide band I refers to the C=O stretching, and the amide band II corresponds to the stretching of the C–N and bending of N–H groups.^38^ In HPI, the amide A and B bands were observed at 3273^–1^ and 2924^–1^ (Figure 2A), while the amide I and II bands were observed at 1637 and 1531 cm^–1^, respectively (Figure 2B). The omnipresent absorption bands observed at 2362 and 2342 cm^–1^ were characteristic of CO_2_^39^ and were ignored in this study.

For NaAlg, three prominent peaks were observed at 1597, 1411, and 1027 cm^–1^. The first two peaks are respectively ascribed to the asymmetric and symmetric stretching vibrations of the COO^–^ groups,^40^ while the latter corresponds to the asymmetric stretching vibration of C–O–C of the pyranic rings of the polysaccharide.^41^

Pure QE crystals showed many specific transmittance peaks which are similar to those discussed by Porto et al.^42^ The broadband with maximum intensity at 3301 cm^–1^ (Figure 2A) corresponds to the stretching of the O–H bonds of the hydroxyl groups present in the aromatic rings. The peak at 1665° (Figure 2B) refers to the C=O carbonyl group. The stretching bonds at 1605, 1559, and 1509 cm^–1^ are characteristic of C=C phenolic bonds. The peak observed at a wavenumber of 1310 cm^–1^ is related to the stretching of =C–O–H groups. The peak with maximum transmittance at 1160 cm^–1^ is ascribed to the stretching of the aromatic ring of the catechol moiety, while the one at 1379 cm^–1^ is tentatively related to the aromatic ring of the other phenol moiety of QE. Finally, the peak at 931 cm^–1^ refers to the bending vibration of C–H bonds.^43^

In the HPI-NaAlg complex spectrum, a slight decrease in the intensity of the functional groups corresponding to HPI (amide A, B, I, and II) was observed. This could point out the presence of interactions with the polysaccharide.^44^ Moreover, the peak at 1411 cm^–1^ in NaAlg related to its COO^–^ functional group disappeared in the HPI-NaAlg complex. This could be due to the high occurrence of ionic interactions between the two polymers at the optimal pH of coacervation and the possible effect of protein excess in the mixture. The latter eventual cause can be observed in the peak at 1027 cm^–1^ (related to the pyranic rings of NaAlg), whose intensity has been significantly reduced but has not completely disappeared (Figure 2B). Changes in the peak at 1597 cm^–1^ of NaAlg (COO^–^ asymmetric stretching) in the mixture cannot be discussed with certainty because it overlapped with that of amide I of the protein, even though it could contribute to the electrostatic interactions.

In QE microcapsules, there was a decrease in the intensity of the characteristic peaks of QE with an increase in the HPI-NaAlg fraction. Particularly, the decrease in the peaks at 3301 cm^–1^ (OH groups) and 1665 cm^–1^ (CO groups) of QE suggests the formation of hydrogen-bound interactions between the latter and the groups of opposite weak electrostatic forces in HPI (e.g., CO and NH, respectively). This affinity between the three elements that composed the HPI-NaAlg-QE complex coacervates obtained at the optimal operating parameters may explain the efficient encapsulation of QE within the HPI-NaAlg coacervates and the high yields of coacervation (Table 1). Furthermore, certain peaks showed slight shifts toward higher wave numbers in the complex coacervates in comparison to the individual components. Particularly, the characteristic band of amide I was shifted from 1637 to 1644 cm^–1^. This band shift was attributed to the establishment of hydrophobic interactions between the polyphenol and the protein within the HPI-NaAlg complex coacervates.^45^ Similar shifts in the amide I region were reported by Ji et al.^46^ upon the formation of complex coacervates between gelatin and sodium carboxymethyl cellulose coencapsulating QE and ascorbic acid. The weakening of the characteristic peak of CH groups in QE (931 cm^–1^)^43^ may constitute another evidence of the occurrence of hydrophobic interactions between QE and the hydrophobic groups in HPI. Hydrophobic interactions between QE and a variety of proteins were evidenced by the literature through both FTIR and fluorescence studies.^47−49^ The following peaks were also upshifted, i.e., the peak corresponding to the asymmetric stretching vibration of C–O–C of the NaAlg pyranic rings (from 1027 to 1034 cm^–1^), the peak corresponding to the stretching of the aromatic ring of the catechol moiety of QE (from 1160 to 1165 cm^–1^), and the characteristic peak of C=C phenolic bonds in QE (from 1509 to 1520 cm^–1^). Wavenumbers may shift due to diverse molecular alterations like bond strength, force constants, and dipole moments.^50^ In the current case, these changes are likely due to the interaction of NaAlg and QE with HPI that led to HPI-NaAlg conservation and the encapsulation of QE within the coacervates.

### 3.4 Thermal Behavior of Coacervates

**Figure 3:**
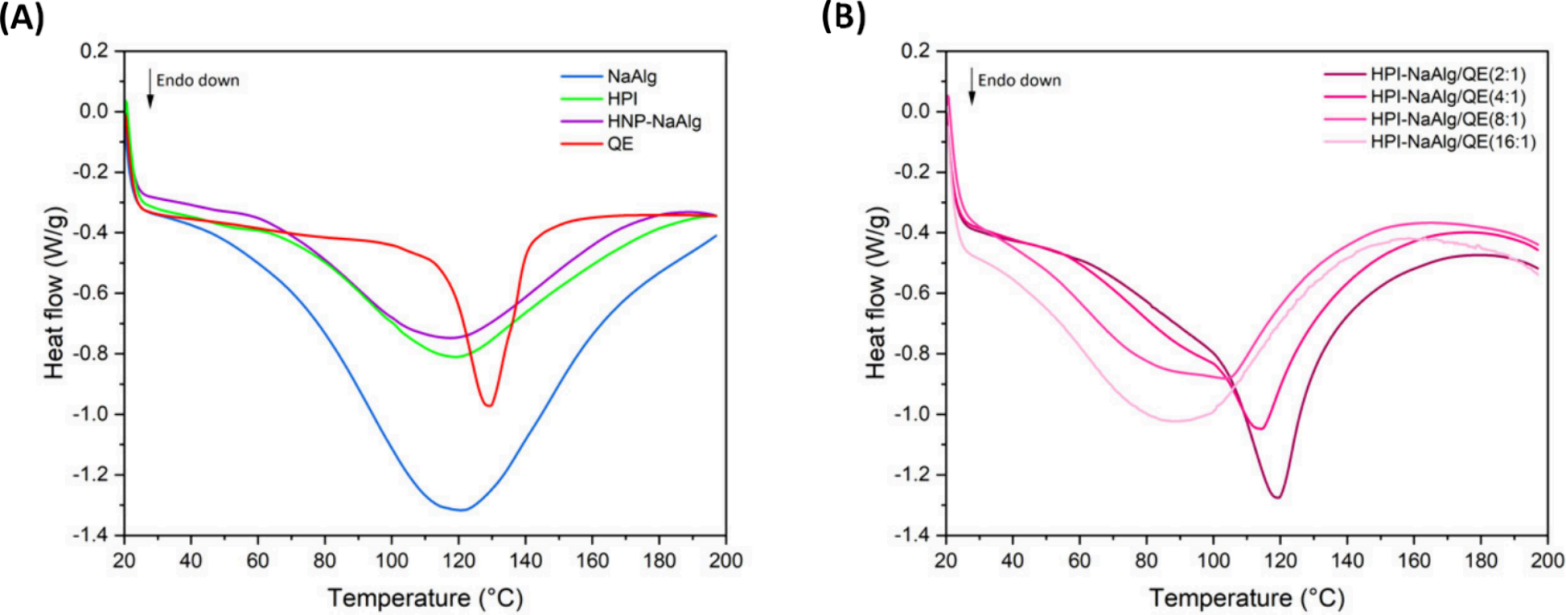
DSC thermograms of sodium alginate (NaAlg), hazelnut protein isolate (HPI), quercetin (QE), HPI-NaAlg coacervates (A), and HPI-NaAlg-QE microcapsules (B).

Differential scanning calorimetry was performed for HPI-NaAlg-QE coacervates and the individual ingredients; the results are presented in Figure 3. All DSC thermograms showed endothermic peaks with variable widths, which indicates a heat uptake process. Both QE and NaAlg peaks correspond to the loss of bound water molecules to form their respective anhydrous form,^51,52^ while the HPI peak is related to the thermal denaturation of the protein.^53^ The intersection of the QE and HPI-NaAlg DSC curves can be easily distinguished in Figure 3B, which indicates a fusion between the two curves. Thus, the maximum heat flow (HF_max_) and the peak temperature (*T*_max_) were preferred over enthalpy (Δ*H*) to discuss Figure 3.

*T*_max_ of QE decreased gradually from 130 °C (Figure 3A) to 119.72, 114.76, and then 104.23 °C in HPI-NaAlg/QE(2:1), HPI-NaAlg/QE(4:1), and HPI-NaAlg/QE(8:1), respectively (Figure 3B). This could be due to a probable facilitation role of HPI-NaAlg in the separation of water molecules from the QE during the dehydration process. The absolute value of HF_max_ also decreased due to the decrease in QE fraction (Figure 3B) but, interestingly, was lower in pure QE (Figure 3A). This could be due to an eventual increase in the QE hydration rate within the HPI-NaAlg coacervates, mainly due to the strong hygroscopicity of NaAlg. Zhang^54^ demonstrated the ability of NaAlg to increase the hygroscopicity of a wheat straw/polylactic acid composite by 40.3% and 69.3% when 10% and 15% of NaAlg were added to the formulations, respectively. In the present study, the NaAlg content in the coacervates is 14.28%.

### 3.5 Microstructure of Coacervates

**Figure 4:**
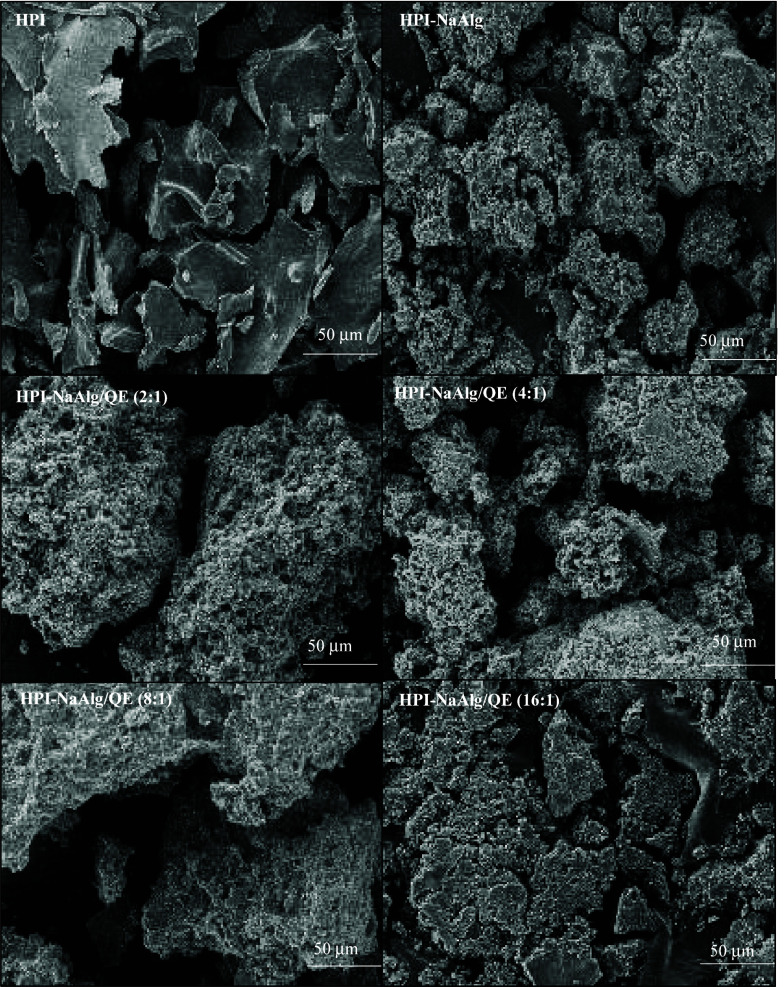
Morphologies of hazelnut protein isolate (HPI), hazelnut protein isolate–sodium alginate coacervates (HPI-NaAlg), and encapsulated quercetin (QE) in HPI-NaAlg with different wall/core ratios.

The surface morphologies of HPI, HPI-NaAlg, and HPI-NaAlg/QE with different core/wall ratios were observed by SEM (Figure 4). There are large heterogeneous clumps of native protein (HPI) with a flakelike structure and smooth surface. The reason for this is likely to be the removal of water during the lyophilization process.^55^ Larger particles and flake-like shapes are likely caused by the absence of forces necessary to break up the frozen liquid into droplets or change their topology significantly during the freezing evaporation process.^56^ Through the interaction between HPI and NaAlg, a porous network structure was formed, interspersed with heterogeneously sized vacuoles. It is possible to include sensitive components in the coacervates via the vacuoles. The sodium-alginate complex containing quercetin preserves the porous structure but exhibits a more ordered block formation. As the quercetin ratio increases, the pore sizes decrease, and the structure becomes more compact.

SEM observations are in accordance with those made by Chen et al.,^57^ who noted that the order displayed on the surface of the coacervate can help stabilize its shape during hollow condensation. Coacervates are structurally ordered, which provides them with stability and functionality. As observed in the SEM images, HPI interacts with NaAlg to form a porous network structure, which is consistent with the study by Croguennec et al.,^58^ in which perfect co-localization of proteins in coacervates has been observed by confocal microscopy and fluorescence resonance energy transfer (FRET) experiments. The SEM analysis of HPI, HPI-NaAlg, and HPI-NaAlg/QE with different core/wall ratios provides valuable insights into the morphological changes induced by the interactions between these components. In the SEM images, distinct structures have been observed to be formed as a result of the presence of NaAlg and quercetin, demonstrating the potential for targeted encapsulation strategies in the food and pharmaceutical industries.

## 4 Conclusions

The objective of this study was to explore a new valorizing application of a large byproduct, hazelnut oil meal. The optimum conditions of complex coacervation formation between hazelnut protein isolate and sodium alginate for the microencapsulation of quercetin were obtained. The results showed that the maximum coacervation occurred at pH 3.5 and a protein/polysaccharide ratio of 6:1, with a coacervation yield as high as 74.9%. HPI-NaAlg complex coacervates achieved a great entrapment efficiency of QE, independently of the wall/core ratio (EE ∼ 99%). FTIR analysis indicated that the coacervation occurred mainly through electrostatic interactions between the amine and amide groups of HPI and the carboxyl groups of NaAlg. Encapsulated QE participated in the structure forming a ternary complex with mainly hydrogen interactions formed between −OH groups within QE and −CO groups of HPI and hydrophobic interactions involving CH groups.

The produced microcapsules can be used as carriers of hydrophobic functional compounds similar to QE in different food matrices. The release of the core material encapsulated within coacervates can be triggered in the intestines, as coacervates are naturally pH-sensitive and prone to proteolysis in the gastrointestinal tract. Nevertheless, there is still a need for the development of an effective method to assess the bioaccessibility of encapsulated fat-soluble compounds as the existing methods require prior emulsification and then the extraction of the compound from the emulsions, which is only suitable for nonencapsulated hydrophobic compounds. Finally, the developed coacervates can be tested and optimized for other bioactive compounds such as essential oils and hydrophilic compounds.

## Supporting Information Available

Visual and microscopic aspects of the coacervates in solution as a function of pH (PDF)
