# Supplementary material for: Hazelnut Protein and Sodium Alginate Complex Coacervates: An Effective Tool for the Encapsulation of the Hydrophobic Polyphenol Quercetin
Source: ACS Omega. 2024 Aug 20;9(35):37243–52. doi: 10.1021/acsomega.4c04859 (PMC11375722; doi:10.1021/acsomega.4c04859)
Supplement: Supplementary file 1 — ao4c04859_si_001.pdf [file ao4c04859_si_001.pdf]

Hazelnut Protein and Sodium Alginate Complex Coacervates: An Effective Tool for the Encapsulation of the Hydrophobic Polyphenol Quercetin

Nabil Adrar[1*], Fatma Duygu Ceylan[1], Esra Capanoglu[1*]

[1]Department of Food Engineering, Faculty of Chemical and Metallurgical Engineering, Istanbul Technical University, Istanbul, Turkey.

Nabil Adrar (adrar@itu.edu.tr)

Fatma Duygu Ceylan (ceylanfa@itu.edu.tr)

Esra Capanoglu (capanogl@itu.edu.tr)

***Corresponding Authors:**

Nabil Adrar

Department of Food Engineering, Faculty of Chemical and Metallurgical Engineering, Istanbul Technical University, Istanbul, Turkey

E-mail: adrar@itu.edu.tr, n.adrar@hotmail.fr,

Esra Capanoglu

Department of Food Engineering, Faculty of Chemical and Metallurgical Engineering, Istanbul Technical University, Istanbul, Turkey

E-mail: capanogl@itu.edu.tr

Supporting Information

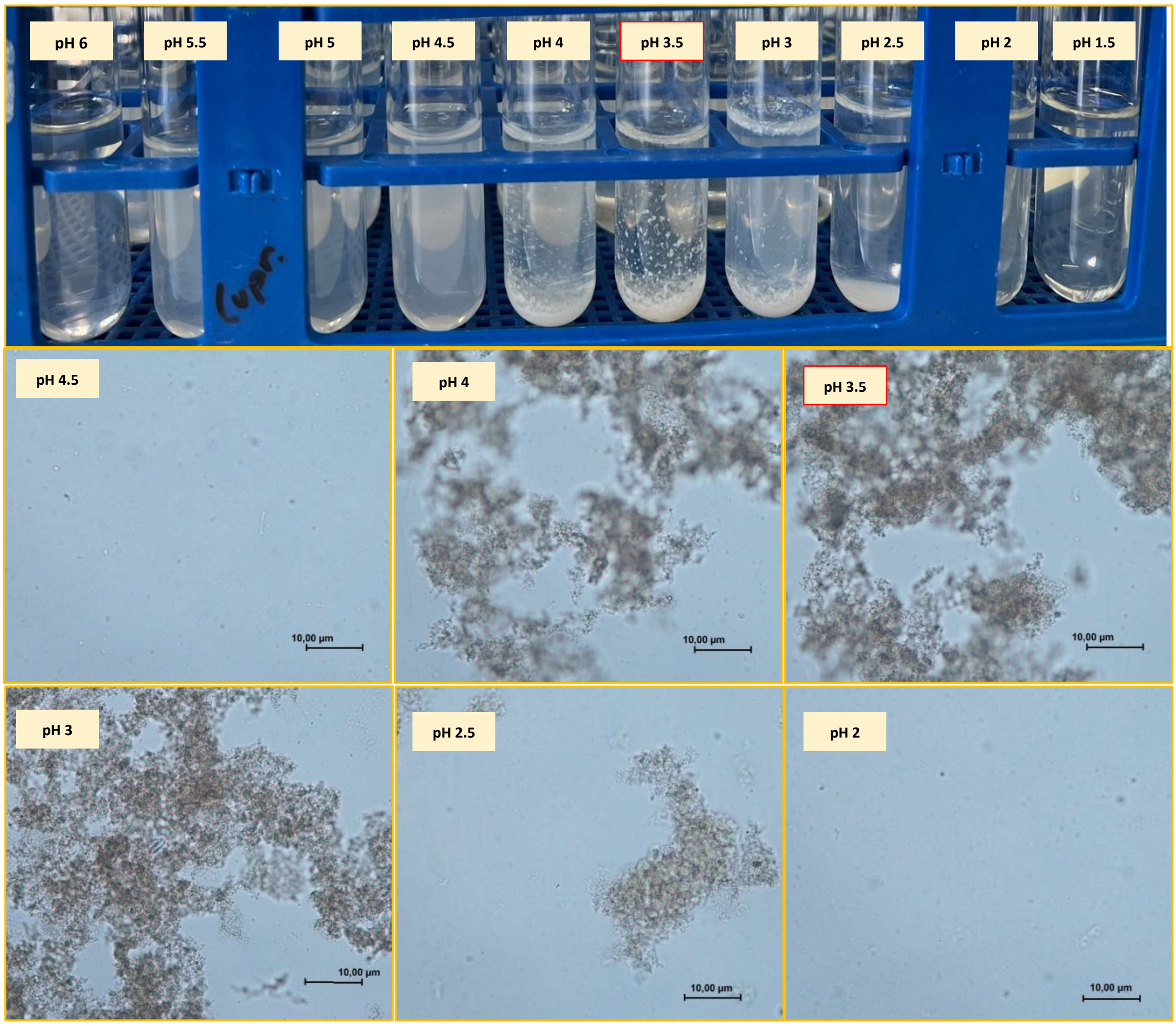

Figure S1. Visual and microscopic aspects of the coacervates in solution as a function of pH.
